# Five-Year Delayed Rupture of a Suspected Infectious Intracranial Aneurysm: A Case Report

**DOI:** 10.7759/cureus.109898

**Published:** 2026-05-29

**Authors:** Mitsuyoshi Abe, Yuki Sakaeyama, Ryo Matsuzaki, Shuhei Kubota, Nobuo Sugo

**Affiliations:** 1 Neurosurgery, Toho University, Tokyo, JPN

**Keywords:** endovascular treatment, infectious intracranial aneurysm, infective endocarditis, intraventricular hemorrhage (ivh), mycotic cerebral aneurysm, parent artery occlusion

## Abstract

Intracranial infectious aneurysms are uncommon cerebrovascular lesions caused by infection-related injury of the arterial wall and may rupture with devastating neurological consequences. A 57-year-old man had developed *Streptococcus mitis* infective endocarditis five years earlier, which was complicated by embolic infarction in the left posterior cerebral artery (PCA) territory and renal infarction, and he had subsequently undergone mitral valve replacement. He presented with sudden impaired consciousness and right hemiplegia while receiving warfarin therapy. Computed tomography (CT) demonstrated hemorrhage in the left PCA territory with intraventricular extension and acute obstructive hydrocephalus. Computed tomography angiography (CTA) showed a contrast spot sign within the hematoma; subsequent cerebral angiography demonstrated a distal left PCA aneurysm. Two sets of blood cultures were negative, and transthoracic echocardiography showed good prosthetic mitral valve motion without apparent vegetation. After anticoagulation reversal and endoscopic intraventricular hematoma evacuation with ventricular drainage, cerebral angiography revealed a 3.4 × 1.7 mm distal left PCA aneurysm. Because severe tortuosity and stenosis of the distal PCA prevented safe catheterization to the aneurysm site, parent artery occlusion was performed using detachable coils from the accessible distal PCA segment. No rebleeding or recurrent hydrocephalus occurred, and the patient was transferred to a long-term care hospital with severe disability. This case illustrates an unusual five-year interval between infective endocarditis-related embolic stroke and delayed rupture of a suspected infectious intracranial aneurysm.

## Introduction

Intracranial infectious aneurysms, historically referred to as mycotic aneurysms, are uncommon cerebrovascular lesions caused by microbial involvement of the arterial wall [1-3]. Infective endocarditis is an important underlying condition, and septic emboli can lodge in distal cerebral arteries, causing arterial wall injury, pseudoaneurysm formation, or rupture [1-3]. Proposed diagnostic criteria for intracranial infectious aneurysms include neuroimaging demonstration of an intracranial aneurysm as a mandatory criterion, together with supportive clinical, microbiological, and imaging features [4].

Treatment is individualized according to rupture status, aneurysm morphology, location, accessibility, systemic infection control, and the patient’s neurological condition [1-3,5]. Antimicrobial therapy may be considered for selected unruptured and stable lesions, whereas ruptured, enlarging, or symptomatic lesions often require surgical or endovascular exclusion from the circulation [1-3,5]. Endovascular parent artery occlusion has been reported as a treatment option for selected distal posterior cerebral artery (PCA) aneurysms when selective aneurysm occlusion or clipping is not feasible [6].

The interval between the onset of infective endocarditis and rupture of an infectious intracranial aneurysm has been reported to range from approximately two to five weeks, and close serial neurovascular imaging has been recommended during this period [7]. Infectious intracranial aneurysms are dynamic lesions; in a cohort of patients managed during antibiotic treatment, unfavorable angiographic or clinical outcomes occurred over a short-term follow-up interval, including aneurysm enlargement, new aneurysm formation, persistence, and rupture [8]. Late rupture after apparent cure of bacterial endocarditis has been described as very unusual [9]. We report a case of intraventricular hemorrhage due to delayed rupture of a distal intracranial aneurysm considered suggestive of an infectious or post-embolic lesion five years after infective endocarditis-related embolic infarction in the same vascular territory.

## Case presentation

A 57-year-old man had developed Streptococcus mitis infective endocarditis five years earlier, which was complicated by embolic infarction in the left temporal-occipital and thalamic regions and renal infarction. For the infective endocarditis, he received ceftriaxone at 2 g twice daily for two weeks before mitral valve replacement, followed by ampicillin (Viccillin) at 2 g twice daily for two weeks after surgery. He had subsequently been receiving warfarin at 3.5 mg/day. There was no recent history of fever, dental treatment, or apparent oral infection. He was transported to our emergency department because of sudden impaired consciousness and right hemiplegia.

At presentation to the emergency department, his Glasgow Coma Scale score was 6 points (E1V1M4) [10]. His body temperature was 36.1°C, pulse rate was 77 beats/min, blood pressure was 126/80 mmHg, and oxygen saturation was 100% with oxygen administered via a face mask. He did not obey verbal commands. His pupils were 1 mm on the right and 3 mm on the left, with a brisk light reflex on the right and a sluggish light reflex on the left. Motor examination showed complete paralysis of the right upper and lower extremities. A withdrawal response was observed in the left upper extremity, and slight movement was observed in the left lower extremity. The National Institutes of Health Stroke Scale score was 40 [11].

Laboratory testing showed a white blood cell count of 9,400/μL and a C-reactive protein level of 0.0 mg/dL, without laboratory evidence of active systemic inflammation. The prothrombin time-international normalized ratio was 2.5 under warfarin therapy, and the D-dimer level was 0.6 μg/mL. Four-factor prothrombin complex concentrate, 1000 IU, and menatetrenone, a vitamin K2 preparation, 20 mg, were administered after arrival for anticoagulation reversal. Two sets of blood cultures were negative.

Transthoracic echocardiography demonstrated preserved left ventricular systolic function. The patient was status post mitral valve replacement, and prosthetic mitral valve opening and closing were good. No obvious vegetation was attached to the prosthetic mitral valve. The left atrium was poorly visualized because of an artifact, whereas right-sided cardiac motion was preserved. Because the patient had no fever, no elevation of C-reactive protein, negative blood cultures, and no apparent vegetation on transthoracic echocardiography, active infective endocarditis was not considered present during the current admission. Antimicrobial therapy directed at active infective endocarditis was therefore not administered. Cefazolin at 1 g twice daily was administered perioperatively for surgical site infection prophylaxis associated with the neurosurgical procedure.

Noncontrast computed tomography (CT) obtained five years earlier showed a low-density area in the left occipital lobe (Figure 1A). Magnetic resonance angiography (MRA) obtained during the same episode showed occlusion of the left PCA, as indicated by the arrow (Figure 1B). Diffusion-weighted magnetic resonance imaging obtained during the same episode showed a hyperintense lesion in the same region as the CT abnormality, consistent with embolic infarction in the left PCA territory (Figure 1C).

**Figure 1:**
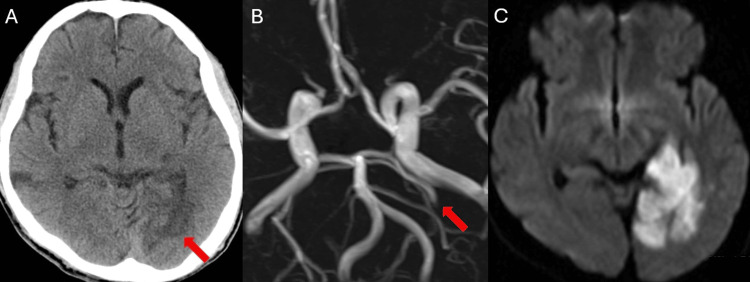
Imaging findings during the previous infective endocarditis-related embolic event (A) Noncontrast CT obtained five years earlier shows a low-density area in the left occipital lobe (arrow). (B) MRA obtained during the same episode shows occlusion of the left PCA, as indicated by the arrow. (C) Diffusion-weighted magnetic resonance imaging obtained during the same episode shows a hyperintense lesion in the same region as the CT abnormality (arrow). CT: computed tomography; MRA: magnetic resonance angiography; DWI: diffusion-weighted imaging; PCA: posterior cerebral artery.

Noncontrast CT at the onset of the current symptoms demonstrated hemorrhage in the left PCA territory, with intraventricular extension and acute obstructive hydrocephalus (Figure 2A). Computed tomography angiography (CTA) obtained at the same time showed a focal contrast spot within the hematoma, compatible with a CTA spot sign rather than a finding diagnostic of an aneurysm itself (Figure 2B) [12]. Reconstructed CTA and venography demonstrated the corresponding enhancing focus within the hematoma (Figure 2C).

**Figure 2:**
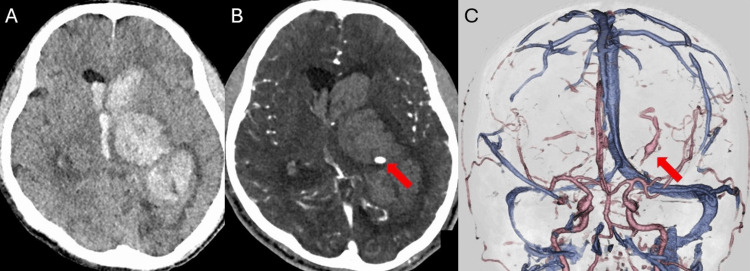
Imaging findings at the onset of the current symptoms (A) Noncontrast CT at the onset of the current symptoms shows hemorrhage in the left PCA territory with intraventricular extension and acute obstructive hydrocephalus. (B) CTA obtained at the same time shows a focal contrast spot within the hematoma, compatible with a CTA spot sign (arrow). (C) Reconstructed CTA and venography demonstrate the corresponding enhancing focus within the hematoma (arrow). CT: computed tomography; CTA: computed tomography angiography; CTV: computed tomography venography; PCA: posterior cerebral artery.

Because of acute obstructive hydrocephalus, endoscopic intraventricular hematoma evacuation with ventricular drainage was performed through the bilateral frontal horns. Postoperative noncontrast CT showed that the tips of the drainage tubes were located within the lateral ventricles, as indicated by the arrowheads (Figure 3A). After stabilization of the patient’s general condition, digital subtraction angiography was performed. Anteroposterior left vertebral angiography demonstrated an aneurysm in the distal left PCA (Figure 3B). Three-dimensional rotational angiography demonstrated a 3.4 × 1.7 mm aneurysm in the distal left PCA territory (Figure 3C). Pre-occlusion angiographic assessment demonstrated that the affected left PCA territory was perfused via leptomeningeal collateral flow from the ipsilateral middle cerebral artery. Given the distal location, hemorrhagic presentation, small aneurysm size, collateral perfusion to the affected territory, and clinical and imaging findings suggestive of an infectious or post-embolic pseudoaneurysmal lesion, endovascular parent artery occlusion was planned. The procedure was performed under local anesthesia via right femoral arterial access. A 5-French ENVOY MPD 90-cm guiding catheter (Codman Neurovascular, Raynham, MA, USA) was placed in the left vertebral artery. Using a Chikai 14 microguidewire (Asahi Intecc Co., Ltd., Aichi, Japan) and an Excelsior SL-10 two-marker preshaped 45° microcatheter (Stryker Neurovascular, Fremont, CA, USA), selective catheterization toward the aneurysm was attempted. However, marked tortuosity and stenosis of the distal PCA prevented advancement of the microcatheter to the aneurysm site. The microcatheter was therefore positioned in the accessible ambient cistern segment of the PCA, and coil embolization was performed as parent artery occlusion. A total of six coils were deployed: one Target 360 Ultra 2 mm × 4 cm coil (Stryker Neurovascular, Fremont, CA, USA), three Axium Prime Helix 1.5 mm × 3 cm coils (Medtronic Neurovascular, Irvine, CA, USA), and two Target Helical Nano 1 mm × 2 cm coils (Stryker Neurovascular, Fremont, CA, USA). Post-treatment anteroposterior left vertebral angiography showed the coil mass in the PCA, as indicated by the arrow, with complete interruption of distal flow beyond the coil mass and disappearance of aneurysmal filling (Figure 3D). Postoperative magnetic resonance imaging was not performed. Follow-up CT did not demonstrate extensive infarction in the left PCA territory. The postoperative course was not complicated by rebleeding or recurrent hydrocephalus. Noncontrast CT at transfer showed no recurrent hemorrhage or recurrent hydrocephalus (Figure 3E). The patient was transferred to a long-term care hospital on hospital day 40 with a modified Rankin Scale score of 5 [13].

**Figure 3:**
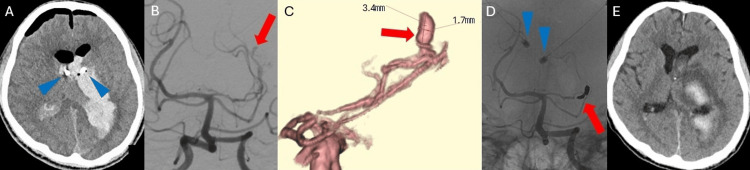
Surgical drainage, angiographic findings, endovascular treatment, and follow-up CT (A) Noncontrast CT after endoscopic intraventricular hematoma evacuation with ventricular drainage through the bilateral frontal horns. The arrowheads indicate the tips of the ventricular drainage tubes within the lateral ventricles. (B) Anteroposterior left vertebral angiography demonstrates an aneurysm in the distal left PCA, as indicated by the arrow. (C) Three-dimensional rotational angiography demonstrates a 3.4 × 1.7 mm distal left PCA aneurysm (arrow). (D) Post-treatment anteroposterior left vertebral angiography shows the coil mass in the PCA, as indicated by the arrow. Distal flow beyond the coil mass is completely interrupted, with the disappearance of aneurysmal filling. The arrowheads indicate the tips of the ventricular drainage tubes. (E) Noncontrast CT at transfer shows no recurrent hemorrhage or recurrent hydrocephalus. CT: computed tomography; DSA: digital subtraction angiography; 3DRA: three-dimensional rotational angiography; PCA: posterior cerebral artery.

## Discussion

This case has three clinically relevant features. First, the aneurysm ruptured five years after the initial infective endocarditis-related embolic event, which is substantially longer than the commonly reported interval of approximately two to five weeks between infective endocarditis onset and infectious intracranial aneurysm rupture [7]. Second, although two sets of blood cultures were negative and transthoracic echocardiography did not show apparent vegetation at the time of hemorrhage, the distal aneurysm location and prior ipsilateral infective endocarditis-related embolic event suggested an infectious or post-embolic aneurysmal mechanism. Third, parent artery occlusion was selected because severe tortuosity and stenosis of the distal PCA prevented safe selective catheterization close to the aneurysm, whereas angiography demonstrated leptomeningeal collateral perfusion to the affected PCA territory from the ipsilateral middle cerebral artery. 

Intracranial infectious aneurysms are thought to occur when septic emboli lodge in distal cerebral arteries and cause infection-related destruction of the arterial wall [1-3]. This process can lead to fusiform dilatation, pseudoaneurysm formation, or rupture [1-3]. These lesions are often located in distal arterial branches, which is consistent with an embolic mechanism [1-3]. In the present case, the patient had previous infective endocarditis caused by Streptococcus mitis, with embolic infarction in the left PCA territory. MRA at that time showed occlusion of the left PCA, and the ruptured aneurysm was later identified in the distal left PCA territory. This chronological and anatomical relationship supports an infection-related or post-embolic mechanism. 

The most unusual aspect of the present case is the long latency between the initial embolic episode and aneurysm rupture. Previous literature indicates that infectious intracranial aneurysm rupture typically occurs in the acute or subacute phase after infective endocarditis onset, with a reported interval of approximately two to five weeks [7]. Rice et al. also described infectious intracranial aneurysms as dynamic lesions during antibiotic treatment, with unfavorable outcomes observed at a median of 21 days, including aneurysm enlargement, new aneurysm formation, persistence, and rupture [8]. In addition, late rupture after apparent cure of bacterial endocarditis has been described as very unusual [9]. Bamford et al. reported rupture of a peripherally located mycotic aneurysm six months after bacteriologically adequate treatment of streptococcal bacterial endocarditis [9], and Terada et al. reported late rupture of a mycotic cerebral aneurysm after mitral valve replacement for bacterial endocarditis in the inactive stage [14]. However, to our knowledge, clearly documented ruptures several years after apparent resolution of infective endocarditis are extremely limited in the PubMed-indexed literature. In contrast to these previously described delayed but shorter courses, the present patient developed hemorrhage five years after the initial infective endocarditis-related embolic infarction. This long interval makes the case distinct from the usual clinical course and suggests that a small infectious or post-embolic aneurysmal lesion may remain clinically silent for a prolonged period before rupture. 

In the present case, the diagnosis of an infectious intracranial aneurysm was not pathologically confirmed. However, the previous Streptococcus mitis infective endocarditis, ipsilateral PCA occlusion at the time of embolic infarction, distal aneurysm location, and later rupture in the same vascular territory were considered collectively suggestive of an infection-related or post-embolic aneurysmal process. Diagnostic criteria for intracranial infectious aneurysms rely on neuroimaging demonstration of an aneurysm and supportive features, but they do not require pathological proof in every case [4]. 

Alternative etiologies of a distal PCA aneurysm include dissecting aneurysm, inflammatory vasculopathy, traumatic or iatrogenic pseudoaneurysm, and a noninfectious de novo distal aneurysm. In the present case, there was no history of recent head trauma or intracranial procedure involving the affected PCA, and there was no clinical or laboratory evidence strongly suggesting active systemic vasculitis at presentation. A dissecting aneurysm could not be completely excluded because pathological confirmation was unavailable. However, the prior Streptococcus mitis infective endocarditis, embolic occlusion of the ipsilateral PCA during the initial episode, and subsequent rupture in the same vascular territory collectively favored an infectious or post-embolic pseudoaneurysmal mechanism. 

At the time of hemorrhage, this patient had no fever, no C-reactive protein elevation, negative blood cultures, and no obvious vegetation on transthoracic echocardiography. These findings argue against active infective endocarditis at presentation. The previous infective endocarditis had been treated with ceftriaxone for two weeks before mitral valve replacement and ampicillin sodium for two weeks after surgery. During the current admission, cefazolin was administered for one week after the neurosurgical procedure solely for surgical site infection prophylaxis and not as treatment for recurrent or persistent infective endocarditis. However, these findings do not exclude the possibility that a small aneurysmal lesion formed during the initial infective endocarditis episode and remained occult or below the detection threshold of noninvasive angiography. Therefore, this case should not be interpreted as evidence that active infection persisted for five years. A more cautious and biologically plausible explanation is that the initial septic embolic event caused chronic arterial wall injury or a small post-embolic pseudoaneurysm, which subsequently remained clinically silent and eventually ruptured. Infection-related damage to the arterial wall, including destruction of the internal elastic lamina and media, may create a structurally fragile segment even after the systemic infection has resolved. Because histopathological confirmation was not available, the lesion should be described as a suspected infectious or post-embolic intracranial aneurysm rather than as a pathologically proven active infectious aneurysm. 

The hemorrhage pattern was compatible with rupture of a distal aneurysmal lesion adjacent to the ventricular system. The patient presented with a left PCA territory hemorrhage with intraventricular extension and acute obstructive hydrocephalus. The focal contrast enhancement on CTA was interpreted as a spot sign, which can indicate active contrast extravasation and hematoma expansion risk, rather than as a definitive diagnostic sign of aneurysm or pseudoaneurysm [12]. Therefore, the initial priority was management of hydrocephalus and intraventricular hematoma, followed by definitive angiographic evaluation and aneurysm treatment after stabilization of the patient’s general condition. 

The management of intracranial infectious aneurysms is individualized [1-3,5]. Medical therapy alone may be considered for selected unruptured and stable lesions, but ruptured, enlarging, or symptomatic lesions often require surgical or endovascular treatment because of the risk of hemorrhage or rebleeding [1-3,5]. Endovascular therapy is an important option for distal infectious aneurysms that are difficult to approach surgically or unsuitable for direct clipping [1,3,5]. 

Selective coil embolization of a small distal infectious or pseudoaneurysmal lesion may be technically difficult and potentially unsafe. In infectious or pseudoaneurysmal lesions, selective coil embolization may be challenging because the aneurysm wall can be fragile, the neck can be poorly defined, and the parent artery can be narrowed or tortuous [1,3,5]. In such situations, parent artery occlusion can be a reasonable deconstructive strategy when the expected ischemic risk is acceptable [1,3,5,6]. In distal PCA aneurysms, parent artery occlusion has been reported as an alternative treatment when selective endovascular treatment or surgical clipping is not suitable [6]. 

In the present case, parent artery occlusion was selected because the distal PCA was markedly tortuous and stenotic, preventing safe microcatheter advancement to the aneurysm. In addition, pre-occlusion angiography showed leptomeningeal collateral perfusion of the affected PCA territory from the ipsilateral middle cerebral artery, which supported the feasibility of a deconstructive strategy. Follow-up CT did not show extensive infarction in the left PCA territory, suggesting that the parent artery occlusion did not result in a large ipsilateral PCA infarction. However, because postoperative magnetic resonance imaging was not performed, small ischemic lesions could not be fully evaluated. 

This report has several limitations. The diagnosis of an infectious intracranial aneurysm was not pathologically confirmed, postoperative magnetic resonance imaging was not performed, and the exact timing of aneurysm formation could not be determined. Although leptomeningeal collateral perfusion to the affected PCA territory was observed angiographically before parent artery occlusion, the collateral flow was not quantitatively evaluated. 

This case should not be interpreted as proof that an active infectious aneurysm persisted for five years. Rather, it suggests that a remote history of infective endocarditis-related embolic stroke may remain clinically relevant when delayed hemorrhage occurs near the previous embolic territory. In such patients, CTA and catheter angiography should be considered to evaluate for a small distal aneurysm, even when inflammatory markers, blood cultures, and echocardiography do not indicate active infection. 

## Conclusions

We report a case of intraventricular hemorrhage due to delayed rupture of a suspected infectious intracranial aneurysm five years after infective endocarditis-related embolic infarction. Although active infection was not demonstrated at the time of hemorrhage and pathological confirmation was unavailable, the distal aneurysm location and prior ipsilateral embolic infarction supported an infection-related or post-embolic pathogenesis. Endovascular parent artery occlusion was feasible when selective catheterization close to the aneurysm was limited by severe distal arterial tortuosity and stenosis, particularly in the presence of leptomeningeal collateral perfusion to the affected PCA territory. This case suggests that delayed rupture of an infectious or post-embolic aneurysmal lesion should remain in the differential diagnosis even years after infective endocarditis when hemorrhage occurs near a previous embolic territory. 
